# Antimicrobial resistance and virulence profiles of staphylococci isolated from clinical bovine mastitis

**DOI:** 10.3389/fmicb.2023.1190790

**Published:** 2023-06-29

**Authors:** Feng Yang, Wenli Shi, Na Meng, Yiyu Zhao, Xuezhi Ding, Qinfan Li

**Affiliations:** ^1^College of Veterinary Medicine, Northwest A&F University, Yangling, Shaanxi, China; ^2^Lanzhou Institute of Husbandry and Pharmaceutical Sciences of Chinese Academy of Agricultural Science, Lanzhou, Gansu, China

**Keywords:** *Staphylococcus aureus*, coagulase-negative staphylococci, antimicrobial resistance, virulence, bovine mastitis

## Abstract

Staphylococci, mainly including *Staphylococcus aureus* and coagulase-negative staphylococci (CNS), are one of the most common pathogens causing bovine mastitis worldwide. In this study, we investigated the antimicrobial resistance and virulence profiles of staphylococci from clinical bovine mastitis in Ningxia Hui Autonomous Region of China. Antimicrobial resistance was determined by disc diffusion combined with *E*-test method. Genes of antimicrobial resistance and virulence factors were determined by PCR. A total of 332 staphylococcal isolates were confirmed from 1,519 mastitic milk samples, including 172 *S. aureus* and 160 CNS isolates. Fifteen CNS species were identified, with *S. chromogenes* being the most frequent found (49.4%), followed by *S. equorum* (13.8%). Noticeably, 2 *S. agnetis* isolates were found among the CNS isolates. To our knowledge, this is the first report documenting the presence of *S. agnetis* from bovine mastitis in China. The *S. aureus* and CNS isolates showed high resistance against penicillin, followed by erythromycin and tetracycline. Multidrug resistance was found in 11.6 and 16.3% of the *S. aureus* and CNS isolates, respectively. Resistance to penicillin was attributed to the presence of *bla*Z, erythromycin resistance to *ermC* (alone or combined with *ermB*) and tetracycline resistance to *tet*K (alone or combined with *tetM*). Notably, one *S. equorum* isolate and one *S. saprophyticus* isolate were both methicillin-resistant and *mecA* positive. Additionally, all *S. aureus* isolates carried the adhesin genes *fnbpA*, *clfA*, *clfB*, and *sdrC*, and most of them contained *cna* and *sdrE*. Conversely, only a few of the CNS isolates carried *clf*A, *cna*, and *fnb*A. Regarding toxin genes, all *S. aureus* isolates harbored *hlb*, and most of them were *hlg* positive. The *lukE*-*lukD*, *lukM*, *sec*, *sed*, *sei*, *sen*, *seo*, *tst*, *seg*, *seh*, and *sej* were also detected with low frequencies. However, no toxin genes were observed in CNS isolates. This study reveals high species diversity of staphylococci from clinical bovine mastitis in Ningxia Hui Autonomous Region of China. The findings for the genetic determinants of antimicrobial resistance and virulence factor provide valuable information for control and prevention of staphylococcal bovine mastitis.

## 1. Introduction

Bovine mastitis remain the most frequent and costly disease affecting dairy cattle due to its effects on health, welfare, and productivity. Staphylococci, mainly including *Staphylococcus aureus* and coagulase-negative staphylococci (CNS), are one of the most common etiological agents causing bovine mastitis worldwide. *S. aureus* is generally considered major mastitis pathogen and mainly induce clinical mastitis, while CNS have traditionally considered minor mastitis-causing pathogen and usually cause subclinical mastitis (Naranjo-Lucena and Slowey, 2023). Currently, however, reports of subclinical and clinical mastitis cases caused by different CNS species have surfaced largely and they have emerged as an important pathogen (Li et al., 2015; De Visscher et al., 2017; Mahato et al., 2017; Ferreira et al., 2022). Among the group of CNS commonly isolated from bovine milk samples, *S. chromogenes*, *S. epidermidis*, *S. haemolyticus*, *S. simulans*, and *S. xylosus* have been identified as the CNS species most likely to cause mastitis (Leroy et al., 2015).

Mastitis is the most common reason for antimicrobials use to control or prevent staphylococcal infections in dairy cattle. Unfortunately, the selective pressure from antimicrobial agents significantly contributes to the dissemination of resistant strains, which greatly attenuate the therapeutic effectiveness of antimicrobial therapy (Isaac et al., 2017; Ahmed et al., 2020). Antimicrobial resistance of staphylococci are mainly attributed to various resistant determinants, such as genes *blaZ* and *mecA* for β-lactams resistance, *tet*s for tetracyclines resistance, and *erm*s for macrolides resistance. The reduced susceptibility of staphylococci against these commonly used antimicrobials in veterinary medicine might promote their persistence in the dairy herd (Kot et al., 2012; Piessens et al., 2012). Therefore, surveillance of antimicrobial resistance is important to ensure optimal results of antimicrobial use and minimize the risk for development and spread of antimicrobial resistance (Waller et al., 2011).

Staphylococci possess a wide variety of virulence factors, including different cell wall-associated adhesins and toxins, that facilitate the bacteria to avoid the immune system and contribute to increased severity of infections. Although most of these factors are originally identified in *S. aureus*, they have also been detected in CNS, including the isolates from bovine origin (González-Martín et al., 2020). In the last decades, the virulence factors in *S. aureus* isolates from bovine mastitis had been frequently reported worldwide. However, despite the emergence of CNS as pathogens, the knowledge regarding their virulence as well antimicrobial resistance in CNS is still poorly understood and is not usually identified at species level, especially the isolates from bovine mastitis in China, which makes it difficult to control infection because a great diversity of species have their own characteristics. Thus, this study was designed to investigate the antimicrobial resistance and virulence profiles of staphylococci isolated from clinical bovine mastitis cases in Ningxia Hui Autonomous Region of China.

## 2. Materials and methods

### 2.1. Bacterial isolation and identification

**Figure 1:**
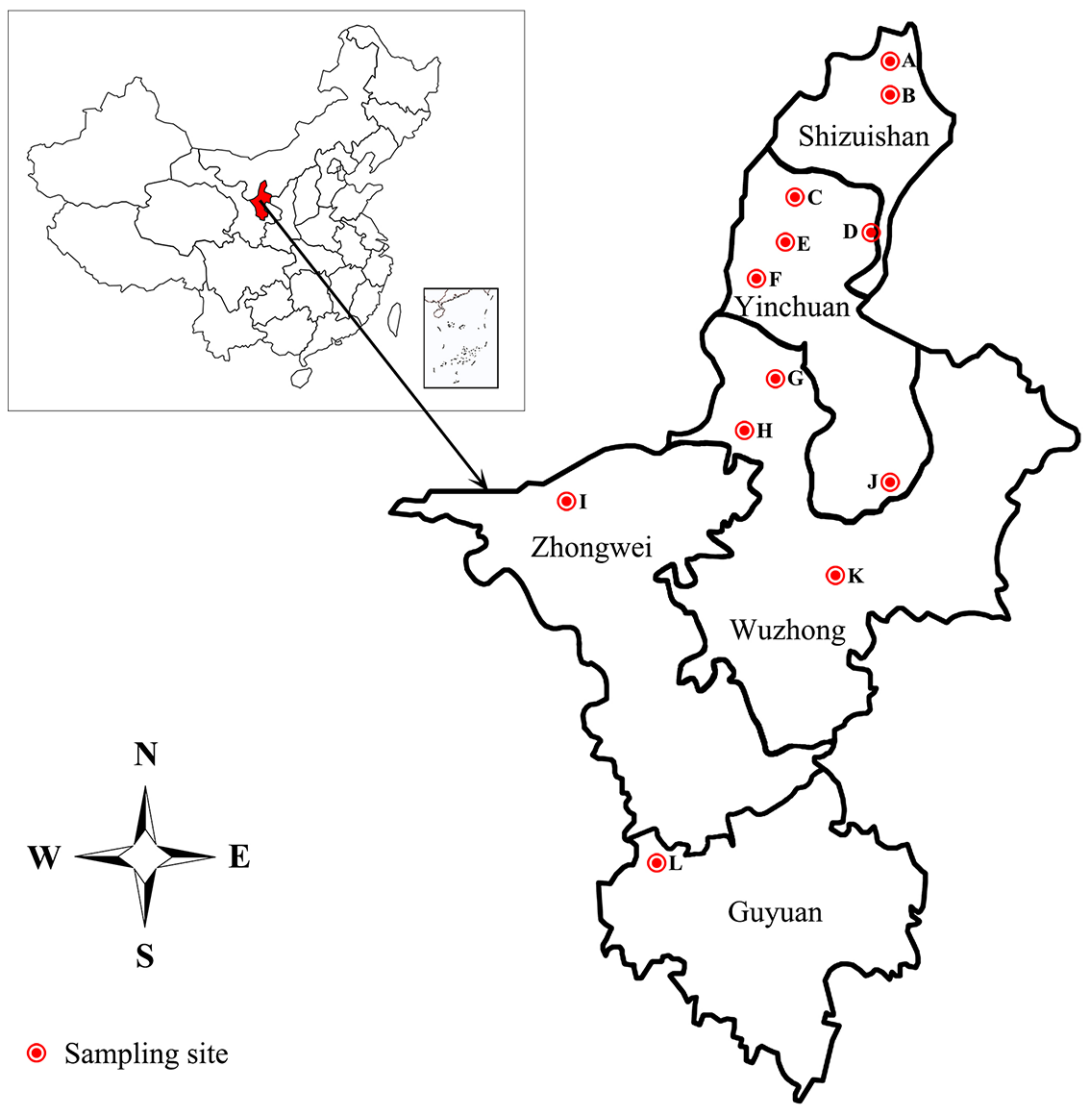
Sampling areas of mastitic milk in Ningxia Hui Autonomous Region, China.

The 332 staphylococcal isolates tested in this study were isolated from 1,519 clinical mastitic milk samples from cows from 12 commercial dairy herds located in Ningxia Hui Autonomous Region in China during July 2021 to Aug 2022 (Figure 1; Supplementary Table S1). Bovine udder showing obvious signs, such as oedema, lumps, increase in temperature, hardening or pain, and milk samples showing any visual evidence of abnormality, such as the presence of clots, flakes or blood, were classified as clinical mastitis (Schmidt et al., 2015; Pérez et al., 2020). Before sampling, teats were disinfected using hydrophilic cotton saturated with 70% ethanol. The first milk squirts were discarded, and 5–10 mL of milk was collected in sterile tubes and transported to the laboratory under refrigeration in cool boxes with ice packs. After transportation to the laboratory, 100 μL of milk was inoculated onto blood agar plates supplemented with 5% defibrinated sheep blood and incubated at 37°C for 48 h. Colonies were initially identified as staphylococcal isolates by appearance (shape, color, and size), Gram staining, catalase and coagulase testing. The suspected isolates were further confirmed by PCR and sequencing as described in our previous study (Yang et al., 2019). Briefly, the genomic DNA was extracted through the Bacterial DNA Kit (Omega Bio-Tek, Norcross, GA) according to the manufacturer’s instructions.^1^ The 16S rRNA gene was amplified by the 16S rDNA Bacterial Identification PCR Kit (Takara, Shiga, Japan) in accordance with the manufacturer’s recommendation.^2^ The PCR products were purified and sequenced by Sanger sequencing by Sangon Biotech (Shanghai) Co., Ltd. in China. Nucleotide sequences were analyzed with the program NCBI-BLAST.^3^ Sequences with 99 to 100% identity to sequences deposited in public domain databases were considered to be positive identification. Confirmed isolates were kept into tryptic soy broth with 20% glycerol at −70°C for molecular testing.

### 2.2. Antimicrobial susceptibility testing

Antimicrobial susceptibility was determined by disc diffusion method on Mueller-Hinton agar (Oxoid, United Kingdom) according to the protocol of Clinical and Laboratory Standards Institute (CLSI, 2018). The panel of antimicrobial agents (Oxoid) included penicillin (10 U), cefoxitin (30 μg), gentamicin (10 μg), erythromycin (15 μg), tetracycline (30 μg), ciprofloxacin (5 μg), nitrofurantoin (300 μg), trimethoprim-sulfamethoxazole (1.25/23.75 μg), chloramphenicol (30 μg), quinupristin/dalfopristin (15 μg), and linezolid (30 μg). Susceptibility to cefoxitin was used to detect the methicillin-resistance phenotype. The *E*-test strips (Liofilchem, Roseto, Italy) were used to detect the vancomycin (0.016 to 256 μg/mL) susceptibility of the staphylococcal isolates. *S. aureus* ATCC 25923 was used as quality control strain. Multidrug-resistant (MDR) isolates were defined as an isolate being resistant to at least 3 antimicrobial agents belonging to different antimicrobial categories (Magiorakos et al., 2012).

### 2.3. Detection of antimicrobial resistance and virulence genes

The resistance genes for penicillin (*blaZ*), methicillin (*mecA* and *mecC*), tetracycline (*tetK* and *tetM*), and erythromycin (*ermA*, *ermB*, and *ermC*) were tested by PCR as described previously using specific primer sets in Supplementary Table S2 (Paterson et al., 2012; Yang et al., 2016). Similarly, the adhesins encoding genes *fnbpA* (fibronectin bind protein), *clfA* and *clfB* (clumping factor), *cna* (collagen binding protein), *sdrC*, *sdrD* and *sdrE* (serine-aspartic acid repeat proteins), *bbp* (bone sialoprotein-binding protein), *ebpS* (elastin-binding protein) and *map*/*eap* (major histocompatibility complex class II analogous protein/extracellular adherence protein), as well as toxins encoding genes *sea*, *seb*, *sec*, *sed*, *see*, *seg*, *seh*, *sei*, *sej*, *sen*, *seo*, and *sem* (staphylococcal enterotoxins), *tst* (toxic shock syndrome toxin-1), *eta* and *etb* (exfoliative toxins), *lukS/lukF-PV*, *lukE*-*lukD*, and *lukM* (leukocidins), *hla*, *hlb*, *hld*, and *hlg* (hemolysins) and *edin* (epidermal cell differentiation inhibitor) were evaluated through PCR (Supplementary Table S2; Jarraud et al., 2002; Peacock et al., 2002). The PCR products were analyzed using 1.0% agarose gel electrophoresis.

## 3. Results

### 3.1. Bacterial identification

Overall, 172 *S. aureus* and 160 CNS isolates were identified from the 332 staphylococcal isolates. Among the CNS isolates, a total of 15 species were identified. The predominant species were *S. chromogenes* (49.4%), followed by *S. equorum* (13.8%), *S. succinus* (9.4%), *S. xylosus* (6.3%), *S. simulans* (5.0%), *S. haemolyticus* (4.4%), *S. hominis* (2.5%), *S. saprophyticus* (1.9%), *S. lugdunensis* (1.9%), *S. gallinarum* (1.9%), *S. agnetis* (1.3%), *S. auricularis* (0.6%), *S. cohnii* (0.6%), *S. epidermidis* (0.6%), and *S. hyicus* (0.6%) (Table 1).

**Table 1:** Distribution and the antimicrobial resistance of staphylococci isolated from clinical bovine mastitis^a^.

Species (No./%)		Antimicrobial resistance (No./%)												MDR (No./%)
		PEN	FOX	VAN	GEN	ERM	TET	CIP	NIT	COT	CHL	QDA	LZD	
*S. aureus*	172/100.0	101/58.7	0/0.0	0/0.0	18/10.5	38/22.1	26/15.1	15/8.7	0/0.0	0/0.0	10/5.8	0/0.0	0/0.0	20/11.6
*S. chromogenes*	79/49.4	60/75.9	0/0.0	0/0.0	2/2.5	25/31.6	14/17.7	1/1.3	0/0.0	0/0.0	2/2.5	0/0.0	0/0.0	12/15.2
*S. equorum*	22/13.8	18/81.8	1/4.5	0/0.0	2/9.1	4/18.2	6/27.3	0/0.0	0/0.0	0/0.0	1/4.5	0/0.0	0/0.0	5/22.7
*S. succinus*	15/9.4	9/60.0	0/0.0	0/0.0	0/0.0	4/26.7	2/13.3	0/0.0	0/0.0	0/0.0	0/0.0	0/0.0	0/0.0	1/6.7
*S. xylosus*	10/6.3	9/90.0	0/0.0	0/0.0	1/10.0	3/30.0	2/20.0	0/0.0	0/0.0	0/0.0	0/0.0	0/0.0	0/0.0	2/20.0
*S. simulans*	8/5.0	5/62.5	0/0.0	0/0.0	4/50.0	2/25.0	1/12.5	1/12.5	0/0.0	0/0.0	0/0.0	0/0.0	0/0.0	1/12.5
*S. haemolyticus*	7/4.4	5/71.4	0/0.0	0/0.0	4/57.1	3/42.9	2/28.6	2/28.6	0/0.0	0/0.0	2/28.6	0/0.0	0/0.0	3/42.9
*S. hominis*	4/2.5	2/50.0	0/0.0	0/0.0	0/0.0	1/25.0	0/0.0	0/0.0	0/0.0	0/0.0	0/0.0	0/0.0	0/0.0	0/0.0
*S. saprophyticus*	3/1.9	2/66.7	1/33.3	0/0.0	1/33.3	1/33.3	1/33.3	0/0.0	0/0.0	0/0.0	1/33.3	0/0.0	0/0.0	1/33.3
*S. lugdunensis*	3/1.9	1/33.3	0/0.0	0/0.0	0/0.0	1/33.3	1/33.3	0/0.0	0/0.0	0/0.0	2/66.7	0/0.0	0/0.0	0/0.0
*S. gallinarum*	3/1.9	1/33.3	0/0.0	0/0.0	0/0.0	0/0.0	0/0.0	0/0.0	0/0.0	0/0.0	0/0.0	0/0.0	0/0.0	0/0.0
*S. agnetis*	2/1.3	1/50.0	0/0.0	0/0.0	0/0.0	1/50.0	1/50.0	0/0.0	0/0.0	0/0.0	0/0.0	0/0.0	0/0.0	0/0.0
*S. auricularis*	1/0.6	0/0.0	0/0.0	0/0.0	0/0.0	0/0.0	0/0.0	0/0.0	0/0.0	0/0.0	0/0.0	0/0.0	0/0.0	0/0.0
*S. cohnii*	1/0.6	0/0.0	0/0.0	0/0.0	0/0.0	0/0.0	0/0.0	0/0.0	0/0.0	0/0.0	0/0.0	0/0.0	0/0.0	0/0.0
*S. epidermidis*	1/0.6	1/100.0	0/0.0	0/0.0	1/100.0	1/100.0	1/100.0	0/0.0	0/0.0	0/0.0	1/100.0	0/0.0	0/0.0	1/100.0
*S. hyicus*	1/0.6	0/0.0	0/0.0	0/0.0	0/0.0	0/0.0	0/0.0	0/0.0	0/0.0	0/0.0	0/0.0	0/0.0	0/0.0	0/0.0
Total CNS	160/100.0	114/71.3	2/1.3	0/0.0	15/9.4	46/28.8	31/19.4	4/2.5	0/0.0	0/0.0	9/7.9	0/0.0	0/0.0	26/16.3

a PEN, penicillin; FOX, cefoxitin; VAN, vancomycin; GEN, gentamicin; ERM, erythromycin; TET, tetracycline; CIP, ciprofloxacin; NIT, nitrofurantoin; COT, trimethoprim-sulfamethoxazole; CHL, chloramphenicol; QDA, quinupristin-dalfopristin; LZD, linezolid.

### 3.2. Antimicrobial susceptibility testing

The antimicrobial susceptibility of the staphylococcal isolates against 12 antimicrobial agents were evaluated. The *S. aureus* isolates showed highest resistance rate to penicillin (101, 58.7%), followed by erythromycin (38, 22.1%), tetracycline (26, 15.1%), gentamicin (18, 10.5%), ciprofloxacin (15, 8.7%), and chloramphenicol (10, 5.8%). In addition, 20 (11.6%) *S. aureus* isolates exhibited MDR. Similar to the antimicrobial resistance profile of *S. aureus*, the CNS isolates displayed high resistance to penicillin (114, 71.3%), followed by erythromycin (46, 28.8%), tetracycline (31, 19.4%), gentamicin (15, 9.4%), chloramphenicol (9, 7.9%), ciprofloxacin (4, 2.5%), and cefoxitin (2, 1.3%). Methicillin-resistant phenotype was detected in 1 *S. equorum* and 1 *S. saprophyticus* isolates based on their susceptibility to cefoxitin. Antimicrobial resistance rates varied by CNS species. Multidrug resistance was found in 26 (16.3%) CNS isolates, including *S. chromogenes* (12, 15.2%), *S. equorum* (5, 22.7%), *S. succinus* (1, 6.7%), *S. xylosus* (2, 20.0%), *S. simulans* (1, 12.5%), *S. haemolyticus* (3, 42.9%), *S. saprophyticus* (1, 33.3%), and *S. epidermidis* (1, 100.0%). None of the staphylococcal isolates showed resistance to nitrofurantoin, trimethoprim-sulfamethoxazole, quinupristin/dalfopristin, linezolid or vancomycin in this study (Table 1, Supplementary Table S3).

### 3.3. Genetic determinants for antimicrobial resistance

The staphylococcal isolates showed higher resistance to penicillin, erythromycin and tetracycline compared to other tested antimicrobial agents in this study. Hence, the resistance encoding genes for these antimicrobial agents as well as methicillin resistant genes *mecA* and *mecC* were tested and shown in Table 2 and Supplementary Table S3. In *S. aureus* isolates, the *blaZ* was detected in 105 (61.0%) isolates. All penicillin-resistant *S. aureus* isolates carried *blaZ*. Besides, 4 penicillin-susceptible isolates also contained this gene. The *tetK* and *tetM* were determined in 21 (12.2%) and 17 (9.9%) isolates, respectively. All *tetK* positive (alone or combined with *tetM*) isolates showed resistance to tetracycline. Five tetracycline-resistant *S. aureus* isolates were negative for *tetK* or *tetM*. Additionally, genes *ermC* and *ermB* were found in 38 (22.1%) and 23 (13.4%) *S. aureus* isolates, respectively. And all erythromycin-resistant isolates harbored *ermC* alone or in combination with *ermB*. None of the isolates were positive for the *mecA*, *mecC* or *ermA*.

**Table 2:** Resistance genes of staphylococci isolated from clinical bovine mastitis.

Species (No.)	Resistance genes (No./%)							
	PEN	OXA		TET		ERM		
	*blaZ*	*mecA*	*mecC*	*tetK*	*tetM*	*ermA*	*ermB*	*ermC*
*S. aureus* (172)	105/61.0	0/0.0	0/0.0	21/12.2	17/9.9	0/0.0	23/13.4	38/22.1
*S. chromogenes* (79)	60/75.9	0/0.0	0/0.0	13/16.5	13/16.9	0/0.0	17/21.5	25/31.6
*S. equorum* (22)	16/72.7	1/4.5	0/0.0	6/27.3	5/22.7	0/0.0	3/13.6	3/13.6
*S. succinus* (15)	9/66.7	0/0.0	0/0.0	2/13.3	1/6.7	0/0.0	1/6.7	4/26.7
*S. xylosus* (10)	9/90.0	0/0.0	0/0.0	2/20.0	0/0.0	0/0.0	1/10.0	3/30.0
*S. simulans* (8)	4/50.0	0/0.0	0/0.0	1/12.5	0/0.0	0/0.0	0/0.0	2/25.0
*S. haemolyticus* (7)	5/71.4	0/0.0	0/0.0	1/14.3	0/0.0	0/0.0	2/28.6	3/42.9
*S. hominis* (4)	2/50.0	0/0.0	0/0.0	0/0.0	0/0.0	0/0.0	0/0.0	1/25.0
*S. saprophyticus* (3)	2/66.7	1/33.3	0/0.0	0/0.0	0/0.0	0/0.0	1/33.3	1/33.3
*S. lugdunensis* (3)	1/33.3	0/0.0	0/0.0	1/33.3	1/33.3	0/0.0	1/33.3	1/33.3
*S. gallinarum* (3)	1/33.3	0/0.0	0/0.0	0/0.0	0/0.0	0/0.0	0/0.0	0/0.0
*S. agnetis* (2)	1/50.0	0/0.0	0/0.0	1/50.0	0/0.0	0/0.0	0/0.0	1/50.0
*S. auricularis* (1)	0/0.0	0/0.0	0/0.0	0/0.0	0/0.0	0/0.0	0/0.0	0/0.0
*S. cohnii* (1)	0/0.0	0/0.0	0/0.0	0/0.0	0/0.0	0/0.0	0/0.0	0/0.0
*S. epidermidis* (1)	1/100.0	0/0.0	0/0.0	1/100.0	0/0.0	0/0.0	1/100.0	1/100.0
*S. hyicus* (1)	0/0.0	0/0.0	0/0.0	0/0	0/0.0	0/0.0	0/0.0	0/0.0
Total CNS (160)	111/69.4	2/1.3	0/0.0	28/17.5	20/12.5	0/0.0	27/16.9	45/28.1

Among the 160 CNS isolates evaluated, the *blaZ* was found in 111 (69.4%) isolates and all of them showed resistance to penicillin. Two *S. equorum* and 1 *S. simulans* that were resistant against penicillin were negative for *blaZ*. Importantly, both of the methicillin-resistant isolates, 1 *S. equorum* and 1 *S. saprophyticus*, carried *mecA*. The *tetK* and *tetM* were determined in 28 (17.5%) and 20 (12.5%) CNS isolates, respectively. All *tetK*-carrying (alone or combined with *tetM*) isolates showed resistance to tetracycline. Three tetracycline-resistant isolates, including 1 *S. chromogenes*, 1 *S. haemolyticus* and 1 *S. saprophyticus*, did not harbored *tetK* or *tetM*. Moreover, *ermC* and *ermB* were detected in 45 (28.1%) and 27 (16.9%) CNS isolates, respectively. All *ermC*-carrying (alone or combined with *ermB*) isolates displayed resistance to erythromycin. One erythromycin-resistant *S. equorum* was negative for *ermC* or *ermB*. None of the CNS isolates were positive for the *mecC* or *ermA* (Table 2, Supplementary Table S3).

### 3.4. Genetic determinants for virulence factors

The presence and distribution of adhesin and toxin genes in staphylococcal isolates were presented in Table 3 and Supplementary Table S3. All *S. aureus* isolates harbored the adhesin genes *fnbpA*, *clfA*, *clfB*, and *sdrC*. Most of them contained *cna* (137, 79.7%) and *sdrE* (118, 68.6%), while genes *ebpS*, *sdrD* and *map*/*eap* were only found in 34.3% (59), 12.8% (22), and 18.6% (32) of the isolates, respectively. For the toxin genes, *hlb* was present in all *S. aureus* isolates, followed by *hlg* (118, 68.6%), *lukE*-*lukD* (59, 34.3%), and *lukM* (38, 22.1%). Genes *sec*, *sed*, *seg*, *seh*, *sei*, *sej*, *sen*, *seo* and *tst* were only observed in 15.7% (27), 15.7% (27), 6.4% (11), 6.4% (11), 15.7% (27), 6.4% (11), 15.7% (27), 15.7% (27), and 15.7% (27) of the isolates, respectively. None of the *S. aureus* isolates were positive for *bbp*, *sea*, *seb*, *see*, *sem*, *eta*, *etb*, *lukS/lukF-PV*, *hla*, *hld*, and *edin*. In contrast to *S. aureus*, virulence genes were detected in a small number of the CNS isolates. Only 10.0% (16) of the CNS isolates were positive for *clfA*, 1.9% (3) for *cna* and 0.6% (1) for *fnbA*. The toxin-encoding genes were not observed in any of the CNS isolates.

**Table 3:** Virulence genes of staphylococci isolated from clinical bovine mastitis.

Virulence genes	Species (No./%)																Total CNS (No./%)
	*S. aureus*	*S. chromogenes*	*S. equorum*	*S. succinus*	*S. xylosus*	*S. simulans*	*S. haemolyticus*	*S. hominis*	*S. saprophyticus*	*S. lugdunensis*	*S. gallinarum*	*S. agnetis*	*S. auricularis*	*S. cohnii*	*S. epidermidis*	*S. hyicus*	
*fnbA*	172/100.0	1/1.3	0/0.0	0/0.0	0/0.0	0/0.0	0/0.0	0/0.0	0/0.0	0/0.0	0/0.0	0/0.0	0/0.0	0/0.0	0/0.0	0/0.0	1/0.6
*clfA*	172/100.0	1/1.3	1/4.6	6/40.0	0/0.0	3/37.5	1/14.3	1/25.0	1/33.3	0/0.0	1/33.3	0/0.0	0/0.0	1/100.0	0/0.0	0/0.0	16/10.0
*clfB*	172/100.0	0/0.0	0/0.0	0/0.0	0/0.0	0/0.0	0/0.0	0/0.0	0/0.0	0/0.0	0/0.0	0/0.0	0/0.0	0/0.0	0/0.0	0/0.0	0/0.0
*cna*	137/79.7	0/0.0	3/13.6	0/0.0	0/0.0	0/0.0	0/0.0	0/0.0	0/0.0	0/0.0	0/0.0	0/0.0	0/0.0	0/0.0	0/0.0	0/0.0	3/1.9
*sdrC*	172/100.0	0/0.0	0/0.0	0/0.0	0/0.0	0/0.0	0/0.0	0/0.0	0/0.0	0/0.0	0/0.0	0/0.0	0/0.0	0/0.0	0/0.0	0/0.0	0/0.0
*sdrD*	22/12.8	0/0.0	0/0.0	0/0.0	0/0.0	0/0.0	0/0.0	0/0.0	0/0.0	0/0.0	0/0.0	0/0.0	0/0.0	0/0.0	0/0.0	0/0.0	0/0.0
*sdrE*	118/68.6	0/0.0	0/0.0	0/0.0	0/0.0	0/0.0	0/0.0	0/0.0	0/0.0	0/0.0	0/0.0	0/0.0	0/0.0	0/0.0	0/0.0	0/0.0	0/0.0
*bbp*	0/0.0	0/0.0	0/0.0	0/0.0	0/0.0	0/0.0	0/0.0	0/0.0	0/0.0	0/0.0	0/0.0	0/0.0	0/0.0	0/0.0	0/0.0	0/0.0	0/0.0
*ebpS*	59/34.3	0/0.0	0/0.0	0/0.0	0/0.0	0/0.0	0/0.0	0/0.0	0/0.0	0/0.0	0/0.0	0/0.0	0/0.0	0/0.0	0/0.0	0/0.0	0/0.0
*map/eap*	32/18.6	0/0.0	0/0.0	0/0.0	0/0.0	0/0.0	0/0.0	0/0.0	0/0.0	0/0.0	0/0.0	0/0.0	0/0.0	0/0.0	0/0.0	0/0.0	0/0.0
*sea*	0/0.0	0/0.0	0/0.0	0/0.0	0/0.0	0/0.0	0/0.0	0/0.0	0/0.0	0/0.0	0/0.0	0/0.0	0/0.0	0/0.0	0/0.0	0/0.0	0/0.0
*seb*	0/0.0	0/0.0	0/0.0	0/0.0	0/0.0	0/0.0	0/0.0	0/0.0	0/0.0	0/0.0	0/0.0	0/0.0	0/0.0	0/0.0	0/0.0	0/0.0	0/0.0
*sec*	27/15.7	0/0.0	0/0.0	0/0.0	0/0.0	0/0.0	0/0.0	0/0.0	0/0.0	0/0.0	0/0.0	0/0.0	0/0.0	0/0.0	0/0.0	0/0.0	0/0.0
*sed*	27/15.7	0/0.0	0/0.0	0/0.0	0/0.0	0/0.0	0/0.0	0/0.0	0/0.0	0/0.0	0/0.0	0/0.0	0/0.0	0/0.0	0/0.0	0/0.0	0/0.0
*see*	0/0.0	0/0.0	0/0.0	0/0.0	0/0.0	0/0.0	0/0.0	0/0.0	0/0.0	0/0.0	0/0.0	0/0.0	0/0.0	0/0.0	0/0.0	0/0.0	0/0.0
*seg*	11/6.4	0/0.0	0/0.0	0/0.0	0/0.0	0/0.0	0/0.0	0/0.0	0/0.0	0/0.0	0/0.0	0/0.0	0/0.0	0/0.0	0/0.0	0/0.0	0/0.0
*seh*	11/6.4	0/0.0	0/0.0	0/0.0	0/0.0	0/0.0	0/0.0	0/0.0	0/0.0	0/0.0	0/0.0	0/0.0	0/0.0	0/0.0	0/0.0	0/0.0	0/0.0
*sei*	27/15.7	0/0.0	0/0.0	0/0.0	0/0.0	0/0.0	0/0.0	0/0.0	0/0.0	0/0.0	0/0.0	0/0.0	0/0.0	0/0.0	0/0.0	0/0.0	0/0.0
*sej*	11/6.4	0/0.0	0/0.0	0/0.0	0/0.0	0/0.0	0/0.0	0/0.0	0/0.0	0/0.0	0/0.0	0/0.0	0/0.0	0/0.0	0/0.0	0/0.0	0/0.0
*sen*	27/15.7	0/0.0	0/0.0	0/0.0	0/0.0	0/0.0	0/0.0	0/0.0	0/0.0	0/0.0	0/0.0	0/0.0	0/0.0	0/0.0	0/0.0	0/0.0	0/0.0
*seo*	27/15.7	0/0.0	0/0.0	0/0.0	0/0.0	0/0.0	0/0.0	0/0.0	0/0.0	0/0.0	0/0.0	0/0.0	0/0.0	0/0.0	0/0.0	0/0.0	0/0.0
*sem*	0/0.0	0/0.0	0/0.0	0/0.0	0/0.0	0/0.0	0/0.0	0/0.0	0/0.0	0/0.0	0/0.0	0/0.0	0/0.0	0/0.0	0/0.0	0/0.0	0/0.0
*tst*	27/15.7	0/0.0	0/0.0	0/0.0	0/0.0	0/0.0	0/0.0	0/0.0	0/0.0	0/0.0	0/0.0	0/0.0	0/0.0	0/0.0	0/0.0	0/0.0	0/0.0
*eta*	0/0.0	0/0.0	0/0.0	0/0.0	0/0.0	0/0.0	0/0.0	0/0.0	0/0.0	0/0.0	0/0.0	0/0.0	0/0.0	0/0.0	0/0.0	0/0.0	0/0.0
*etb*	0/0.0	0/0.0	0/0.0	0/0.0	0/0.0	0/0.0	0/0.0	0/0.0	0/0.0	0/0.0	0/0.0	0/0.0	0/0.0	0/0.0	0/0.0	0/0.0	0/0.0
*lukS/lukF-PV*	0/0.0	0/0.0	0/0.0	0/0.0	0/0.0	0/0.0	0/0.0	0/0.0	0/0.0	0/0.0	0/0.0	0/0.0	0/0.0	0/0.0	0/0.0	0/0.0	0/0.0
*lukE-lukD*	59/34.3	0/0.0	0/0.0	0/0.0	0/0.0	0/0.0	0/0.0	0/0.0	0/0.0	0/0.0	0/0.0	0/0.0	0/0.0	0/0.0	0/0.0	0/0.0	0/0.0
*lukM*	38/22.1	0/0.0	0/0.0	0/0.0	0/0.0	0/0.0	0/0.0	0/0.0	0/0.0	0/0.0	0/0.0	0/0.0	0/0.0	0/0.0	0/0.0	0/0.0	0/0.0
*hla*	0/0.0	0/0.0	0/0.0	0/0.0	0/0.0	0/0.0	0/0.0	0/0.0	0/0.0	0/0.0	0/0.0	0/0.0	0/0.0	0/0.0	0/0.0	0/0.0	0/0.0
*hlb*	172/100.0	0/0.0	0/0.0	0/0.0	0/0.0	0/0.0	0/0.0	0/0.0	0/0.0	0/0.0	0/0.0	0/0.0	0/0.0	0/0.0	0/0.0	0/0.0	0/0.0
*hld*	0/0.0	0/0.0	0/0.0	0/0.0	0/0.0	0/0.0	0/0.0	0/0.0	0/0.0	0/0.0	0/0.0	0/0.0	0/0.0	0/0.0	0/0.0	0/0.0	0/0.0
*hlg*	118/68.6	0/0.0	0/0.0	0/0.0	0/0.0	0/0.0	0/0.0	0/0.0	0/0.0	0/0.0	0/0.0	0/0.0	0/0.0	0/0.0	0/0.0	0/0.0	0/0.0
*edin*	0/0.0	0/0.0	0/0.0	0/0.0	0/0.0	0/0.0	0/0.0	0/0.0	0/0.0	0/0.0	0/0.0	0/0.0	0/0.0	0/0.0	0/0.0	0/0.0	0/0.0

## 4. Discussion

A variety of bacteria have been implicated in bovine mastitis, with staphylococci being considered one of the most significant and prevalent causative agents in China and other countries (Gao et al., 2017). Understanding the pathogen profile for mastitis is critical to management (Dyson et al., 2022). In routine mastitis diagnostic laboratories, CNS are usually not identified to the species level but are reported as a single group. Consequently, limited knowledge is available regarding the epidemiology and relative importance of different species in this group (Ruegg et al., 2015; Schmidt et al., 2015; Dyson et al., 2022; Zigo et al., 2022). Although a protective effect against clinical mastitis has been postulated (Addis et al., 2020), ascribing the beneficial effect to the CNS as a group is probably inaccurate and still a topic of debate; such effect will rather be situated at the species or even strain level (Vanderhaeghen et al., 2014). The CNS group isolated from bovine milk samples consists of more than 50 different species and subspecies (Locatelli et al., 2013), and the distribution of CNS species change over time and vary between different regions (Dyson et al., 2022). In our study, 172 *S. aureus* and 160 CNS isolates were identified from the 332 staphylococcal isolates through 16S rRNA gene sequencing. A total of 15 species were confirmed among the CNS isolates. These species were frequently observed in both clinical and subclinical mastitis with slight differences among herds worldwide (Frey et al., 2013; Condas et al., 2017; El-Razik et al., 2017; Lianou et al., 2021), but the proportion of different *Staphylococcus* species varied between studies carried out in different countries (Schmidt et al., 2015; Xu et al., 2015). In accordance with the previous reports (Rall et al., 2014; Dos Santos et al., 2016; Valckenier et al., 2021), the predominant CNS species analyzed in this study was *S. chromogenes*. Normally, *S. equorum* was a less frequently detected species among CNS from dairy cattle (Adkins et al., 2018; Mahmmod et al., 2018; Jenkins et al., 2019; Valckenier et al., 2021). However, the *S. equorum* was the second most prevalent CNS species in our study, similar to the report that high proportion of this species was found in bulk milk (De Visscher et al., 2017), suggesting that this species might be relevant for udder health in the sampling site. Additionally, although *S. epidermidis* and *S. haemolyticus* commonly presents a high prevalence among CNS from bovine origin (Lee and Lee, 2022), the *S. epidermidis* and *S. haemolyticus* in our study were only observed in few isolates. This may indicate that these two species are not significant causative agent of mastitis in our studied area. Management practices, origin and strategy of samples, housing systems, climate and herd size used in the studies could probably explain some of the differences. Moreover, the distribution of the most common species has been shown to change over time (Koop et al., 2012; Nyman et al., 2018). Notably, although the prevalence was low, *S. agnetis* isolates were identified among the CNS isolates in the current study. This *Staphylococcus* species, an emerging pathogen, was described as a separate species in 2012 and frequently isolated from mastitic milk samples in other countries (Condas et al., 2017; Mahato et al., 2017; Poulsen et al., 2017; Rahmdel et al., 2018; Szafraniec et al., 2020). To our knowledge, this is the first report documenting the occurrence of *S. agnetis* from bovine mastitis in China. Further sampling is required to ascertain the true prevalence and significance of this species in local dairy herds.

Antimicrobial therapy has been used as a successful strategy for controlling staphylococcal mastitis. β-Lactams, tetracyclines and macrolides were commonly used to treat staphylococcal mastitis. But the therapeutic effects are hampered by the increasing number of drug-resistant strains (Kim et al., 2019; Achek et al., 2020). In the present study, the most resistance was observed against penicillin in both *S. aureus* and CNS isolates, followed by erythromycin and tetracycline. Meanwhile, low resistance rates of gentamicin, ciprofloxacin and chloramphenicol were also found in the staphylococcal isolates tested in this study. Resistance to these antimicrobials was also frequently reported by other authors (Cheng et al., 2019; Vasileiou et al., 2019; Francisco et al., 2021; Lianou et al., 2021; Mostafa Abdalhamed et al., 2022). In agreement with other recent studies (Fernandes Dos Santos et al., 2016; Taponen et al., 2016), resistance to the tested antimicrobials was higher in CNS than that in *S. aureus* with the exception of gentamicin and ciprofloxacin in the present study. Nevertheless, our results were similar to previous studies reporting low-level resistance to gentamicin and ciprofloxacin in both *S. aureus* and CNS isolates from bovine mastitis (Frey et al., 2013; Mahato et al., 2017; Martins et al., 2017; Klibi et al., 2018), probably due to the low frequent use of these antimicrobials in dairy farm in comparison with penicillin, erythromycin and tetracycline. Notably, 1 *S. equorum* and 1 *S. saprophyticus* isolates were resistant to methicillin in the current study. The occurrence of methicillin resistance in these 2 CNS species isolated from humans, livestock and farm environment has been previously described (Cicconi-Hogan et al., 2014; Teeraputon et al., 2017; Lu et al., 2020; Bonvegna et al., 2021; Garbacz et al., 2021). However, to the best of the available knowledge, there are no reports of the methicillin resistance in *S. equorum* and *S. saprophyticus* causing bovine mastitis in China. These resistant bacteria have been reported as an emerging problem in veterinary medicine and pose a threat to public health due to their transfer from animals to the humans caring for them (Kim et al., 2019). Moreover, our findings were in accord with previous study found that CNS often exhibit greater tendency to develop multidrug resistance (MDR) than *S. aureus* (Schmidt et al., 2015). The high phenotypic resistance could be explained by the frequent use of these antimicrobials for the treatment of mastitis or other diseases such as lameness, respiratory, or reproductive problems. An augmented exposure to antimicrobials can lead to an increase in resistant strains and consequently to the diversity we observed in the resistance profile of the isolates (Fernandes Dos Santos et al., 2016; Osman et al., 2016). Furthermore, we found very large differences in antimicrobial resistance between different CNS species, possibly due to the limited number of isolates at the species level.

In this study, the most commonly antimicrobial resistance determined was against penicillin, erythromycin and tetracycline. Thus, the genes conferring resistance to these antimicrobials as well as methicillin were detected. Corresponding to the phenotypic resistance, *blaZ* showed high prevalence and was found in all penicillin-resistant *S. aureus* isolates in this study. However, 4 *blaZ*-containing isolates were susceptible to penicillin. Previous studies also found the phenomenon that some *blaZ*-positive *S. aureus* isolates were susceptible to penicillin (Ruegg et al., 2015; Andrade et al., 2021). The discrepancy may be attributable to the lack of *blaZ* expression (Hammad et al., 2014). In CNS, all *blaZ*-positive isolates were resistant to penicillin. But 3 penicillin-resistant CNS isolates were negative for this gene. This may be attributed to the fact that mechanisms such as efflux pump or biofilm other than expression of the *blaZ* gene can cause penicillin resistance because multiple mechanisms of resistance often exist in these isolates (Osman et al., 2015; Addetia et al., 2019; Francisco et al., 2021). Moreover, the *mecA* was observed in both of the methicillin-resistant isolates (one isolate each of *S. equorum* and *S. saprophyticus*), which confirmed the phenotypic resistance to methicillin. To date, at least 38 tetracycline resistance genes have been found, and the genes *tetK* and *tetM* has been commonly found in species of staphylococci (Ruegg et al., 2015). In this study, despite the low occurrence of *tetM*, the *tetK* was determined in most of the tetracycline-resistant staphylococcal isolates. This may not be surprising because *tetK* is very frequent in staphylococci species from cows with clinical mastitis (Klibi et al., 2018). Furthermore, all *tetK*-carrying (alone or combined with *tetM*) isolates showed resistance to tetracycline. A few staphylococcal isolates showed phenotypic resistance to tetracycline but were negative for *tetM* or *tetK*. Additionally, 10 genes have been identified encoding resistance to erythromycin until now, being *ermA*, *ermB*, and *ermC* the major mechanism in staphylococci for erythromycin resistance (Sun et al., 2018). But in our study, aside from 1 erythromycin-resistant *S. equorum* that was negative for *ermC* or *ermB*, the *ermC* alone or in combination with *ermB* were detected in all erythromycin-resistant staphylococcal isolates, which is supported by previous research indicating that *ermC* is the most prevalent *erm* gene recovered from cases of staphylococcal bovine mastitis and most of the isolates exhibited phenotypic resistance to erythromycin (Ruegg et al., 2015). The coexistence of these tetracyclines and macrolides resistance genes has been frequently reported in *S. aureus* or CNS isolates from bovine mastitis in China and other countries (Klibi et al., 2018; Naranjo-Lucena and Slowey, 2023). The discrepancies observed between the phenotypic susceptibility and resistance genes could be due to the presence of other resistance-encoding genes, such as *tetL* or *tetO* for tetracyclin and *ermE*, *ermT*, *mefA*, or *mefE* for erythromycin, or due to a mutation in the primer-annealing site (DiPersio et al., 2008; Schmidt et al., 2015).

The pathogenicity of staphylococci is mainly related to its capacity to encode and produce a multitude of virulence factors, facilitating their adhesion and invasion of the host cells and establishment of infection (Klibi et al., 2018). The initial attachment of staphylococci to epithelial cells of the teat canal depends on the interaction of bacterial adhesins with host surface proteins, peptides and molecules located in the basement membrane (Stutz et al., 2011). In the current study, the *S. aureus* isolates exhibited high prevalence of *fnbpA*, *clfA*, *clfB*, *sdrC*, *cna*, and *sdrE*. Similar observations have also been reported in our previous study and by other reports (Yang et al., 2020; Avila-Novoa et al., 2022; Ibrahim et al., 2022). However, the frequencies of *ebpS*, *sdrD*, and *map*/*eap* found in our study was lower than those reported by other authors (Cheraghi et al., 2017; Kot et al., 2022). Our findings indicated that a diversity of adhesins were involved in the initial attachment of host cells and colonization of the mammary gland by *S. aureus* in Ningxia Hui Autonomous Region. In addition, this group of isolates was also evaluated for toxin genes related to the invasion of host cells and the evasion of immune response. Hemolysins are pore-forming toxins that attack cell membranes and cause platelet damage, lysosome destruction, ischemia, and necrosis (Abril et al., 2020). Most *S. aureus* isolates from bovine and human origins have been reported to primarily possess the *hla*, which causes incomplete or partial hemolysis (Zhang et al., 2018; Khan et al., 2021). However, the *hlb* and *hlg* were the predominant hemolysin genes in this study, and none of the isolates contained *hla*. Leukocidins are also pore-forming two-component toxins that specifically attack immune cells (Abril et al., 2020). Similar to previous studies (Haveri et al., 2007, 2008; Thomas et al., 2021), *lukE*-*lukD* was the most prevalent leukocidin-encoding gene in our study, followed by *lukM*. Moreover, enterotoxins and toxic shock syndrome toxin-1 are pyrogenic toxins known as staphylococcal superantigens causing staphylococcal food poisoning and are able to interrupt host immune responses (Podkowik et al., 2013; Abril et al., 2020). In the present study, enterotoxin-encoding genes *sec*, *sed*, *seg*, *seh*, *sei*, *sej*, *sen*, and *seo* as well as toxic shock syndrome toxin-1-encoding gene *tst* were detected with low frequencies. These findings were in accordance with those of other reports involved in bovine *S. aureus* (Hummerjohann et al., 2014; Rall et al., 2014; Mello et al., 2016 Vaughn et al., 2020). The variation in the prevalence of the tested virulence factors could be associated with the genetic diversity of strains, the source and sizes of samples or their geographic locations (Avila-Novoa et al., 2022). Given that certain virulence genes are overrepresented in some clonal lineages and that some combinations are correlated with high pathogenic potential (Achek et al., 2020), further investigations need to be performed to explore the diversity of virulence factors combination in *S. aureus* pathogenesis.

Consistent with other studies (Supré et al., 2011; Xu et al., 2015; França et al., 2021), the virulence genes in CNS were significantly less prevalent than that in the *S. aureus* in our study. Previous study indicated that collagen binding protein (*cna*) and fibronectin binding protein (*fnbA*) were often associated with CNS attachment in bovines (Pizauro et al., 2019). However, in the current study, only few of the CNS isolates carried *cna* and *fnbA*. Similar results were obtained from subclinical mastitis milk in China (Xu et al., 2015). Additionally, a few of the CNS isolates were positive for *clfA* in this study, in line with the report by Felipe et al. that 12.2% of the CNS isolates contained this gene (Felipe et al., 2017). The capacity to adhere to bovine mammary epithelial cells strongly differs among the different CNS isolates and potentially reflects intra-species diversity in ecology and epidemiological behavior (Souza et al., 2016). Recent studies have provided strong evidence for the presence of toxin genes and production of the corresponding toxins in CNS in China and other countries, especially the enterotoxins (Rall et al., 2014; Salaberry et al., 2015; Mahato et al., 2017; Martins et al., 2017; Pizauro et al., 2019). However, the toxin-encoding genes were not observed in any of the CNS isolates in our study. Our results were similar to those presented by other authors that all CNS species were negative for the toxin genes, even though a wide range of genes were tested (Nemati et al., 2008; Klempt et al., 2022). This may be attributed to the fact that the low prevalence of toxin-producing CNS isolates in the studied area, or the presence of other toxin genes which were not tested (Xu et al., 2015). Another possible reason is the requirement of different primer sets to detect the target genes (Vanderhaeghen et al., 2014). Furthermore, previous studies demonstrated that the use of antimicrobial drugs influenced the expression of virulence genes in staphylococci. The connection between genetic elements conferring resistance to antimicrobials and expression of virulence factors is intricately linked to the ability of bacteria to communicate through two-component system and quorum sensing system and has not yet been fully elucidated (Pérez et al., 2020). In further research, large sample size and sufficient numbers of isolates of each species are needed to explore the species-specific association between antimicrobial resistance and virulence factors in staphylococci.

## 5. Conclusion

This study provides high species diversity of staphylococci from clinical bovine mastitis in Ningxia Hui Autonomous Region in China. Noticeably, to our knowledge, we first describe the occurrence of *S. agnetis* from bovine mastitis in China. The *S. aureus* and CNS isolates displayed high frequencies of phenotypic and genotypic resistance to penicillin, erythromycin and tetracycline, which remind the government to pay continuous attention to the commonly used antimicrobial agents in dairy industry. Moreover, the high occurrence of the adhesin genes *fnbpA*, *clfA*, *clfB*, *sdrC*, *cna*, and *sdrE* tested in this study as well as the toxin genes *hlb* and *hlg* in *S. aureus* indicate their pathogenic potential causing bovine mastitis in the studied area. Further investigation is necessary to explore the diversity of virulence factors combination in *S. aureus* pathogenesis. Furthermore, despite the absence of toxin genes in CNS in this study, a more extensive examination is needed to demonstrate the true toxigenic potential of this organisms group in mastitis.

## Data availability statement

The original contributions presented in the study are included in the article/Supplementary material, further inquiries can be directed to the corresponding authors.

## Ethics statement

The animal study was reviewed and approved by Animal Welfare and Ethics Committee of Northwest A&F University. Written informed consent was obtained from the owners for the participation of their animals in this study.

## Author contributions

FY conceptualized the study, designed the methodology, conducted the tests, and wrote the original paper. WS, NM, and YZ helped to conduct the experimental tests. XD and QL provided resources, made the review, as well as were the leadership and responsible for funding acquisition. All authors contributed to the article and approved the submitted version.

## Funding

This work was financially supported by the Key Research and Development Program of Gansu Province (Grant No. 21YF5NA141) and the National Key R&D Program of China during the 14th Five-year Plan Period (Grant No. 2022YFD1302101).

## Conflict of interest

The authors declare that the research was conducted in the absence of any commercial or financial relationships that could be construed as a potential conflict of interest.

## Publisher’s note

All claims expressed in this article are solely those of the authors and do not necessarily represent those of their affiliated organizations, or those of the publisher, the editors and the reviewers. Any product that may be evaluated in this article, or claim that may be made by its manufacturer, is not guaranteed or endorsed by the publisher.

## Footnotes

^1^https://www.omegabiotek.com/product/e-z-n-a-bacterial-dna-kit/

^2^https://www.takarabiomed.com.cn/DownLoad/RR176.pdf

^3^http://www.ncbi.nlm.nih.gov
