# Supplementary material for: Antimicrobial resistance and virulence profiles of staphylococci isolated from clinical bovine mastitis
Source: Front Microbiol. 2023 Jun 29;14:1190790. doi: 10.3389/fmicb.2023.1190790 (PMC10344457; doi:10.3389/fmicb.2023.1190790)
Supplement: Supplementary file 1 [file Table_1.DOCX]

***Supplementary Materials***

**Antimicrobial resistance and virulence profiles of staphylococci isolated from clinical bovine mastitis**

**Feng Yang^1,2^, Wenli Shi^1^, Na Meng^1^, Yiyu Zhao^1^, Xuezhi Ding^2*^, Qinfan Li^1*^**

*** Correspondence:** Qinfan Li, [liqf1131@163.com](mailto:liqf1131@163.com,); Xuezhi Ding, [dingxuezhi@caas.cn](mailto:dingxuezhi@caas.cn).

**Supplementary Table S1.** Information of mastitic milk sampling and bacterial isolation

| Herds | Cows (No.) | Milk samples (No.) | Isolates (No.) | *S. aureus* (No.) | CNS (No.) |
| --- | --- | --- | --- | --- | --- |
| A | 285 | 285 | 296 | 62 | 36 |
| B | 334 | 334 | 339 | 22 | 45 |
| C | 20 | 20 | 19 | 9 | 2 |
| D | 94 | 94 | 86 | 14 | 23 |
| E | 101 | 101 | 107 | 11 | 7 |
| F | 36 | 36 | 33 | 1 | 6 |
| G | 27 | 27 | 28 | 5 | 1 |
| H | 22 | 22 | 23 | 1 | 4 |
| I | 31 | 31 | 31 | 0 | 2 |
| J | 61 | 61 | 62 | 2 | 2 |
| K | 403 | 403 | 417 | 31 | 22 |
| L | 105 | 105 | 112 | 14 | 10 |
| Total | 1519 | 1519 | 1553 | 172 | 160 |

**Supplementary Table S2.** Primers and positive control used in this study.

| Target gene | Sequence(5'-3') | Product size (bp) | Annealing temperature (°C) | Positive control | Reference |
| --- | --- | --- | --- | --- | --- |
| *blaZ* | F: TAAGAGATTTGCCTATGCTT | 373 | 48 | ATCC 33591 | Yang et al., 2016 |
|  | R: TTAAAGTCTTACCGAAAGCAG |  |  |  |  |
| *mecA* | F: TGGTATGTGGAAGTTAGATTGGGAT | 155 | 60 | ATCC 33591 | Yang et al., 2016 |
|  | R: CTAATCTCATATGTGTTCCTGTATTGGC |  |  |  |  |
| *mecC* | F: CATTAAAATCAGAGCGAGGC | 188 | 57 | ATCC BBA-2312 | Paterson et al., 2012 |
|  | R: TGGCTGAACCCATTTTTGAT |  |  |  |  |
| *tetK* | F: GTAGCGACAATAGGTAATAGT | 360 | 48 | *S. aureus*^b^ | Yang et al., 2016 |
|  | R: GTAGTGACAATAAACCTCCTA |  |  |  |  |
| *tetM* | F: AGTGGAGCGATTACAGAA | 158 | 48 | *S. aureus*^b^ | Yang et al., 2016 |
|  | R: CATATGTCCTGGCGTGTCTA |  |  |  |  |
| *ermA* | F: TCTAAAAAGCATGTAAAAGAA | 645 | 45 | RN1389 | Yang et al., 2016 |
|  | R: CTTCGATAGTTTATTAATATTAGT |  |  |  |  |
| *ermB* | F: GAAAAGGTACTCAACCAAATA | 639 | 45 | *S. aureus*^b^ | Yang et al., 2016 |
|  | R: AGTAACGGTACTTAAATTGTTTAC |  |  |  |  |
| *ermC* | F: TCAAAACATAATATAGATAAA | 642 | 45 | RN4220 | Yang et al., 2016 |
|  | R: GCTAATATTGTTTAAATCGTCAAT |  |  |  |  |
| *fnbA* | F: CACAACCAGCAAATATAG | 1362 | 52 | ATCC 29213 | Peacock et al., 2002 |
|  | R: CTGTGTGGTAATCAATGTC |  |  |  |  |
| *clfA* | F: GTAGGTACGTTAATCGGTT | 1584 | 55 | Newman | Peacock et al., 2002 |
|  | R: CTCATCAGGTTGTTCAGG |  |  |  |  |
| *clfB* | F: TGCAAGATCAAACTGTTCCT | 596 | 55 | Newman | Peacock et al., 2002 |
|  | R: TCGGTCTGTAAATAAAGGTA |  |  |  |  |
| *cna* | F: AGTGGTTACTAATACTG | variable^a^ | 46 | ATCC 25923 | Peacock et al., 2002 |
|  | R: CAGGATAGATTGGTTTA |  |  |  |  |
| *sdrC* | F: ACGACTATTAAACCAAGAAC | 560 | 55 | Newman | Peacock et al., 2002 |
|  | R: GTACTTGAAATAAGCGGTTG |  |  |  |  |
| *sdrD* | F: GGAAATAAAGTTGAAGTTTC | 500 | 52 | Newman | Peacock et al., 2002 |
|  | R: ACTTTGTCATCAACTGTAAT |  |  |  |  |
| *sdrE* | F: CAGTAAATGTGTCAAAAGA | 767 | 50 | Sanger 476 | Peacock et al., 2002 |
|  | R: TTGACTACCAGCTATATC |  |  |  |  |
| *bbp* | F: CAGTAAATGTGTCAAAAGA | 1055 | 50 | NRS71 | Peacock et al., 2002 |
|  | R: TACACCCTGTTGAACTG |  |  |  |  |
| *ebpS* | F: CAATCGATAGACACAAATTC | 526 | 53 | NRS71 | Peacock et al., 2002 |
|  | R: CAGTTACATCATCATGTTTA |  |  |  |  |
| *map/eap* | F: TAACATTTAATAAGAATCAA | 946 | 46 | Newman | Peacock et al., 2002 |
|  | R: CCATTTACTGCAATTGT |  |  |  |  |
| *sea* | F: GAAAAAAGTCTGAATTGCAGGGAACA | 560 | 60 | ATCC 13565 | Jarraud et al., 2002 |
|  | R: CAAATAAATCGTAATTAACCGAAGGTTC |  |  |  |  |
| *seb* | F: ATTCTATTAAGGACACTAAGTTAGGGA | 404 | 60 | ATCC 14458 | Jarraud et al., 2002 |
|  | R: ATCCCGTTTCATAAGGCGAGT |  |  |  |  |
| *sec* | F: GTAAAGTTACAGGTGGCAAAACTTG | 297 | 60 | ATCC 19095 | Jarraud et al., 2002 |
|  | R: CATATCATACCAAAAAGTATTGCCGT |  |  |  |  |
| *sed* | F: GAATTAAGTAGTACCGCGCTAAATAATATG | 492 | 60 | ATCC 8095 | Jarraud et al., 2002 |
|  | R: GCTGTATTTTTCCTCCGAGAGT |  |  |  |  |
| *see* | F: CAAAGAAATGCTTTAAGCAATCTTAGGC | 482 | 60 | ATCC 27664 | Jarraud et al., 2002 |
|  | R: CACCTTACCGCCAAAGCTG |  |  |  |  |
| *seg* | F: AATTATGTGAATGCTCAACCCGATC | 642 | 60 | ATCC 27661 | Jarraud et al., 2002 |
|  | R: AAACTTATATGGAACAAAAGGTACTAGTTC |  |  |  |  |
| *seh* | F: CAATCACATCATATGCGAAAGCAG | 376 | 60 | ATCC 51811 | Jarraud et al., 2002 |
|  | R: CATCTACCCAAACATTAGCACC |  |  |  |  |
| *sei* | F: CTCAAGGTGATATTGGTGTAGG | 576 | 60 | ATCC 27661 | Jarraud et al., 2002 |
|  | R: AAAAAACTTACAGGCAGTCCATCTC |  |  |  |  |
| *sej* | F: TAACCTCAGACATATATACTTCTTTAACG | 300 | 60 | ATCC 8095 | Jarraud et al., 2002 |
|  | R: AGTATCATAAAGTTGATTGTTTTCATGCAG |  |  |  |  |
| *sen* | F: ATGAGATTGTTCTACATAGCTGCAAT | 680 | 60 | ATCC 27661 | Jarraud et al., 2002 |
|  | R: AACTCTGCTCCCACTGAAC |  |  |  |  |
| *seo* | F: AGTTTGTGTAAGAAGTCAAGTGTAGA | 180 | 60 | ATCC 27661 | Jarraud et al., 2002 |
|  | R: ATCTTTAAATTCAGCAGATATTCCATCTAAC |  |  |  |  |
| *sem* | F: CTATTAATCTTTGGGTTAATGGAGAAC | 300 | 60 | ATCC 27661 | Jarraud et al., 2002 |
|  | R: TTCAGTTTCGACAGTTTTGTTGTCAT |  |  |  |  |
| *tst* | F: TTCACTATTTGTAAAAGTGTCAGACCCACT | 180 | 60 | FRI 1169 | Jarraud et al., 2002 |
|  | R: TACTAATGAATTTTTTTATCGTAAGCCCTT |  |  |  |  |
| *eta* | F: ACTGTAGGAGCTAGTGCATTTGT | 190 | 60 | CCM7056 | Jarraud et al., 2002 |
|  | R: TGGATACTTTTGTCTATCTTTTTCATCAAC |  |  |  |  |
| *etb* | F: CAGATAAAGAGCTTTATACACACATTAC | 612 | 60 | CCM7056 | Jarraud et al., 2002 |
|  | R: AGTGAACTTATCTTTCTATTGAAAAACACTC |  |  |  |  |
| *lukS/lukF-PV* | F: ATCATTAGGTAAAATGTCTGGACATGATCCA | 433 | 60 | ATCC 49775 | Jarraud et al., 2002 |
|  | R: GCATCAASTGTATTGGATAGCAAAAGC |  |  |  |  |
| *lukE-lukD* | F: TGAAAAAGGTTCAAAGTTGATACGAG | 269 | 60 | FRI 913 | Jarraud et al., 2002 |
|  | R: TGTATTCGATAGCAAAAGCAGTGCA |  |  |  |  |
| *lukM* | F: TGGATGTTACCTATGCAACCTAC | 780 | 60 | ATCC 31890 | Jarraud et al., 2002 |
|  | R: GTTCGTTTCCATATAATGAATCACTAC |  |  |  |  |
| *hla* | F: CTGATTACTATCCAAGAAATTCGATTG | 209 | 60 | FRI 913 | Jarraud et al., 2002 |
|  | R: CTTTCCAGCCTACTTTTTTATCAGT |  |  |  |  |
| *hlb* | F: GTGCACTTACTGACAATAGTGC | 309 | 60 | NCTC 7428 | Jarraud et al., 2002 |
|  | R: GTTGATGAGTAGCTACCTTCAGT |  |  |  |  |
| *hld* | F: AAGAATTTTTATCTTAATTAAGGAAGGAGTG | 111 | 60 | NCTC 9393 | Jarraud et al., 2002 |
|  | R: TTAGTGAATTTGTTCACTGTGTCGA |  |  |  |  |
| *hlg* | F: GTCAYAGAGTCCATAATGCATTTAA | 535 | 60 | ATCC 49775 | Jarraud et al., 2002 |
|  | R: CACCAAATGTATAGCCTAAAGTG |  |  |  |  |
| *edin* | F: GAAGTATCTAATACTTCTTTAGCAGC | 625 | 60 | E 1 | Jarraud et al., 2002 |
|  | R: TCATTTGACAATTCTACACTTCCAAC |  |  |  |  |

^a^Variable product size depending on the number of B repeats (multiples of ~560 nucleotides).

^b^Verified field isolate in our previous study.

**References**

Jarraud, S., Mougel, C., Thioulouse, J., Lina, G., Meugnier, H., Forey, F., et al. (2002). Relationships between *Staphylococcus aureus* genetic background, virulence factors, *agr* groups (alleles), and human disease. *Infect. immun*. 70, 631-641. doi: 10.1128/IAI.70.2.631-641.2002

Paterson, G. K., Larsen, A. R., Robb, A., Edwards, G. E., Pennycott, T. W., Foster, G., et al. (2012). The newly described *mecA* homologue, mecALGA251, is present in methicillin-resistant *Staphylococcus aureus* isolates from a diverse range of host species. *J. Antimicrob. Chemother*. 67, 2809-2813. doi: 10.1093/jac/dks329

Peacock, S. J., Moore, C. E., Justice, A., Kantzanou, M., Story, L., Mackie, K., et al. (2002). Virulent combinations of adhesin and toxin genes in natural populations of *Staphylococcus aureus.* *Infect. immun*. 70, 4987-4996. doi: 10.1128/IAI.70.9.4987-4996.2002

Yang, F., Wang, Q., Wang, X., Wang, L., Li, X., and Luo, J., et al. (2016). Genetic characterization of antimicrobial resistance in *Staphylococcus aureus* isolated from bovine mastitis cases in northwest china. *J. Integr. Agric*. 15, 2842-2847. doi: 10.1016/S2095-3119(16)61368-0
